# Usefulness of a hub and spoke TDM-guided expert clinical pharmacological advice program of dalbavancin for optimizing very long-term curative or suppressive treatment of chronic staphylococcal infections

**DOI:** 10.1128/aac.01830-24

**Published:** 2025-02-24

**Authors:** Pier Giorgio Cojutti, Milo Gatti, Sara Tedeschi, Eleonora Zamparini, Marianna Meschiari, Maria Danzi, Giacomo Menegotto, Marco Cotrufo, Laura Soavi, Erika Chiari, Marco Ripa, Maria Mazzitelli, Massimo Crapis, Annamaria Cattelan, Giustino Parruti, Alessandro Russo, Lorenzo Zammarchi, Carlo Tascini, Pierluigi Viale, Federico Pea

**Affiliations:** 1 Department of Medical and Surgical Sciences, Alma Mater Studiorum, University of Bologna 198207 , Bologna, Italy; 2 Clinical Pharmacology Unit, IRCCS Azienda Ospedaliero-Universitaria di Bologna, Bologna, Italy; 3 Infectious Diseases Unit, Department for Integrated Infectious Risk Management, IRCCS Azienda Ospedaliero-Universitaria di Bologna, Bologna, Italy; 4 Department of Surgery, Medicine, Dentistry and Morphological Sciences, University of Modena and Reggio Emilia 198220 , Modena, Italy; 5 Unit of Infectious Diseases, Santa Chiara Hospital, APSS 18701 , Trento, Italy; 6 Infectious Diseases Clinic, Santa Maria della Misericordia University Hospital of Udine, ASUFC 18707 , Udine, Italy; 7 UOC Malattie Infettive, ASST Papa Giovanni XXIII 9333 , Bergamo, Italy; 8 Division of Infectious and Tropical Diseases, ASST Spedali Civili 18515 , Brescia, Italy; 9 Infectious Diseases Unit, Vita-Salute San Raffaele University 18985 , Milan, Italy; 10 Infectious Diseases Unit, IRCCS San Raffaele Scientific Institute, Milan, Italy; 11 Infectious and Tropical Diseases Unit, Padua University Hospital 18624 , Padua, Italy; 12 Infectious Diseases Unit, S. Maria degli Angeli Hospital, Pordenone, Italy; 13 Infectious Diseases Unit, Pescara General Hospital, Pescara, Italy; 14 Department of Medical and Surgical Sciences, "Magna Graecia" University 335566 , Catanzaro, Italy; 15 Infectious and Tropical Disease Unit, "Renato Dulbecco" Teaching Hospital, Catanzaro, Italy; 16 Department of Experimental and Clinical Medicine, University of Florence 415681 , Florence, Italy; 17 Department of Infectious and Tropical Diseases, Azienda Ospedaliero Universitaria Careggi 18561 , Florence, Italy; 18 Department of Medical Area, University of Udine 9316 , Udine, Italy; Providence Portland Medical Center, Portland, Oregon, USA

**Keywords:** dalbavancin, therapeutic drug monitoring, model-informed precision dosing, long-term treatment

## Abstract

A hub and spoke model for optimizing long-term treatment of chronic staphylococcal infections with dalbavancin based on therapeutic drug monitoring (TDM)-guided expert clinical pharmacological advice (ECPA) was implemented. This multicentric retrospective cohort study included patients receiving dalbavancin monotherapy lasting >6 weeks at different spoke hospitals having treatment optimized by means of a TDM-guided ECPA program at a hub hospital. Optimal pharmacokinetic/pharmacodynamic target against staphylococci with an MIC up to 0.125 mg/L was defined as dalbavancin concentrations >8.04 mg/L. Patients received dalbavancin therapy for curative (curative group) or suppressive (suppressive group) purposes. Clinical outcome was assessed by means of repeated ambulatory visits. A total of 12 spoke hospitals applied for 414 TDM-based ECPA for 101 patients, of whom 64.4% (65/101) were treated for curative and 35.6% (36/101) were for suppressive purposes. In the curative and suppressive groups, TDM-based ECPA optimized treatment for up to 14 and 28 months, respectively, and ensured median optimal exposure of 95.7% and 100%, respectively. In the curative group, having <70% of treatment time with concentrations above the optimal target increased failure risk [odds ratio (OR), 6.71; confidence interval (CI), 0.97–43.3; *P* = 0.05]. In the suppressive group, infective endocarditis was associated with an increased risk of ineffective treatment (OR, 8.65; CI, 1.29–57.62; *P* = 0.046). Mild adverse events were reported in 4.5% (5/101) of cases. A hub and spoke TDM-guided ECPA program of dalbavancin may be cost-effective for optimizing long-term treatment of chronic staphylococcal infections and for patients admitted to hospitals lacking in-house MD clinical pharmacologists.

## INTRODUCTION

Dalbavancin is a novel long-acting lipoglycopeptide with activity against most Gram-positive bacteria including multidrug-resistant staphylococci (1). Although it is licensed as a single 1,500 mg dose for treating acute bacterial skin and skin structure infections covering up to 14 days (1–3), its off-label use for long-term treatment of chronic infections is ever growing nowadays. This is favored by its cost-effective features in terms of very long elimination half-life, valuable tissue penetration, and good safety profile (4, 5).

The most frequent types of off-label indications of dalbavancin are bone and joint infections (BJI), prosthetic joint infections (PJI), infective endocarditis, vascular graft infections, and other Gram-positive biofilm-related infections (6). Frequently, these types of infections are characterized by a chronic course, and in most cases, several weeks or even months of antimicrobial treatment are needed for definitive microbiological eradication, sometimes coupled with surgical debridement. In some other cases, source control is unfeasible so that long-term suppressive antibiotic therapy must be adopted for preventing bacterial regrowth over time (7, 8). A recent study reviewed the four most common clinical indications requiring long-term antibiotic use for suppression of bacterial infections, namely, PJI, vascular graft infections, cardiac implantable electronic device infections, and osteomyelitis and spinal hardware infections (9). Interestingly, patients failing primary anti-infective treatment and those not eligible for a surgical approach because of underlying frailty or comorbidities emerged as the two most prevalent scenarios for adopting this strategy. Beta-lactams and tetracyclines were the most commonly prescribed antibiotics, and the rates of adverse events as high as 41–52% were reported, especially for the latter (9).

When using dalbavancin for long-term treatment of chronic staphylococcal infections, optimal exposure in terms of pharmacokinetic/pharmacodynamic (PK/PD) target attainment against staphylococci should be maintained over time. The optimal target corresponds to a free 24 h area under the concentration-time curve to MIC (*f*AUC_24h_/MIC) ratio > 111.1 (10) and may be granted over time only by administering multiple re-dosing. In this regard, we showed previously that keeping in patients total dalbavancin plasma concentrations above 8.04 mg/L over time may be helpful for this purpose. This threshold may ensure an optimal PK/PD target attainment against all of the dalbavancin-susceptible staphylococcal strains up to the EUCAST clinical breakpoint, namely, those with an MIC value of 0.125 mg/L (10, 11). Based on this assumption, we showed by means of a population pharmacokinetic coupled with Monte Carlo simulation that two 1,500 mg dalbavancin doses administered 1 week apart on day 1 and day 8 may predict optimal PK/PD target attainment for up to 6 weeks in the vast majority of patients with osteoarticular infections (12).

However, whenever dalbavancin treatment should last longer, it is almost impossible to choose *a priori* an appropriate additional dosing regimen because the inter-individual pharmacokinetic variability of dalbavancin may be quite high (12, 13). In these latter cases, therapeutic drug monitoring (TDM) has been advocated as a valuable tool for estimating both the duration of optimal PK/PD target attainment and the timing for proper re-dosing (11, 13). A recent international consensus of experts agreed that TDM should be especially advantageous in clinical scenarios characterized by treatments lasting longer than 6 weeks and should be ideally started in the timeframe between day 21 and day 35 after administering a cumulative dose of 3,000 mg in the first 2–3 weeks (14).

At our tertiary university hospital, a TDM-guided expert clinical pharmacological advice (ECPA) program led by MD clinical pharmacologists was established for optimizing long-term therapy with dalbavancin of subacute and chronic staphylococcal infections (15). A recent proof-of-concept study carried out among patients with chronic staphylococcal osteoarticular infections showed that this approach could allow a very high rate of successful clinical outcome (16). Consequently, we recently implemented a model-informed precision dosing (MIPD) Bayesian approach for guiding more precisely the TDM-guided ECPA, thus improving as much as possible the accuracy of the program (17).

On these bases and thanks to the peculiar long-acting features of dalbavancin, a hub and spoke collaboration has been implemented between our tertiary university hospital and other hospitals across Italy by offering the opportunity of optimizing long-term treatment with dalbavancin even in patients admitted to other hospitals lacking in-house PK/PD specialist properly managing TDM programs, namely, MD clinical pharmacologists or ID clinical pharmacists.

The aim of this study was to assess the usefulness of this hub and spoke model of TDM-guided ECPA of dalbavancin in optimizing very long-term curative and/or suppressive treatment of chronic staphylococcal infections.

## RESULTS

**Fig 1:**
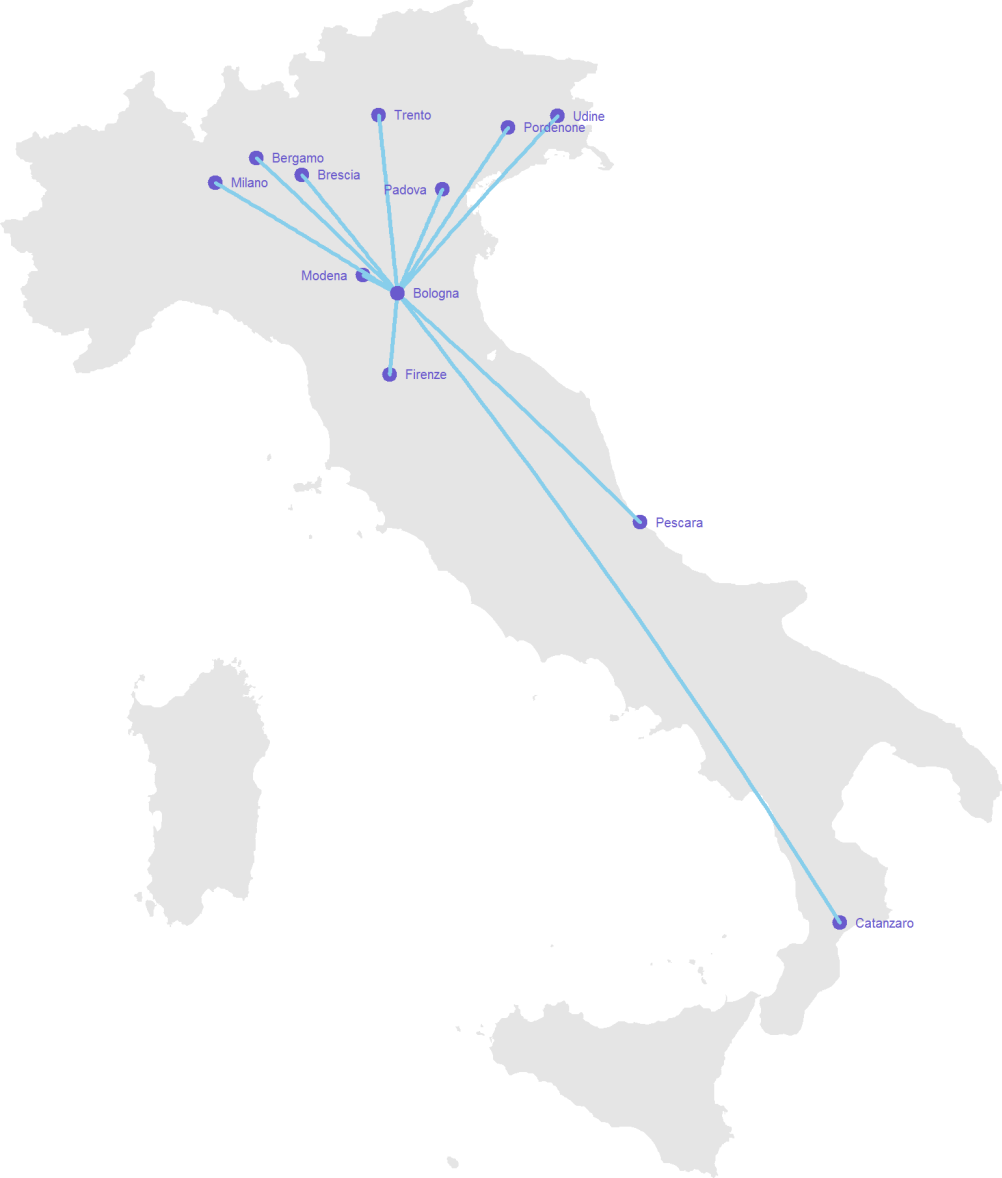
Connection map of the hub and spoke model showing Italian hospitals joining the TDM-guided expert clinical pharmacological advice program for optimizing dalbavancin long-term therapy. The map was created using the libraries “maps” and “geosphere” of R software.

**Fig 2:**
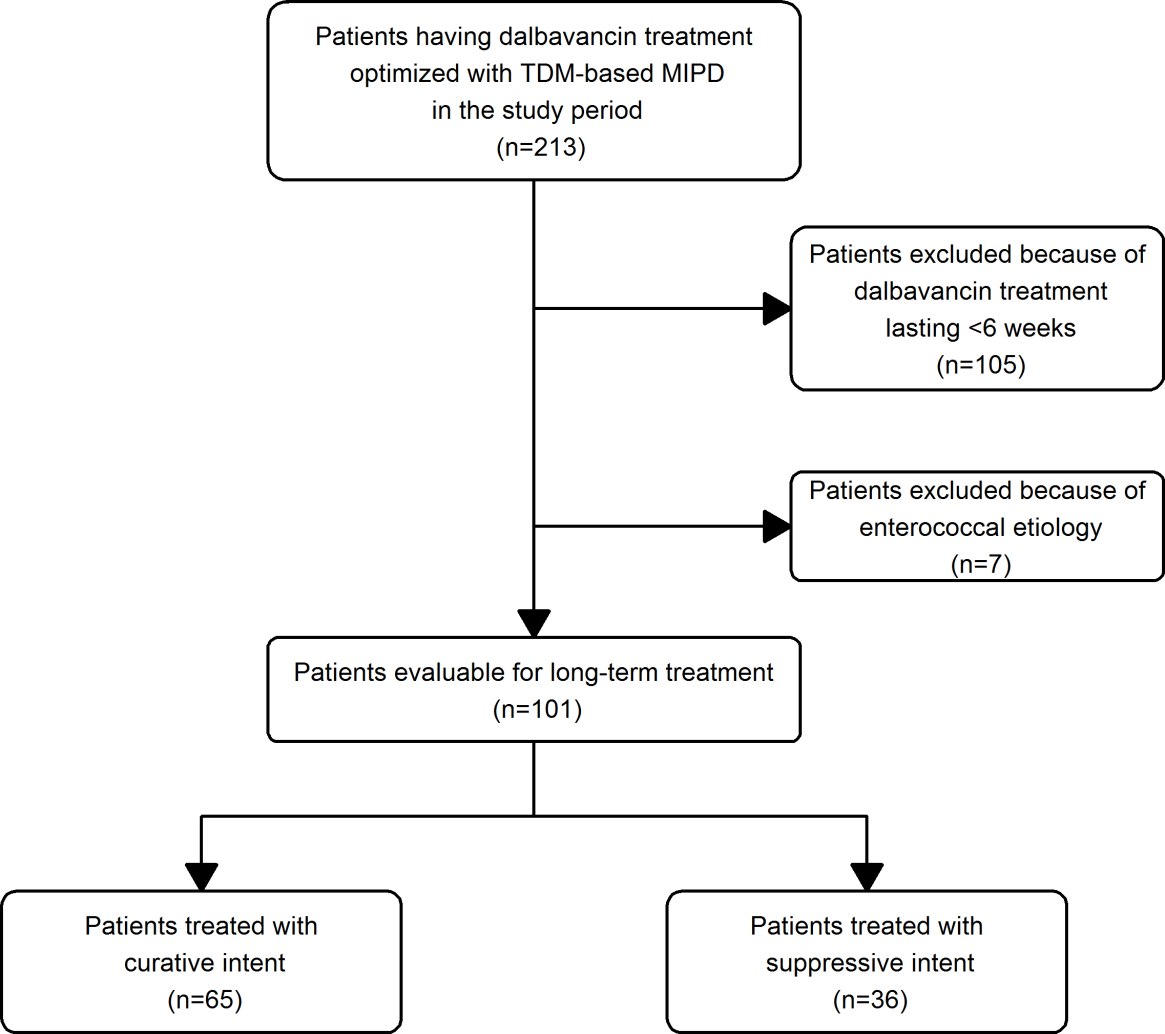
Flowchart of patient inclusion criteria in the study.

A total of 12 hospitals joined the program (1 hub plus 11 spokes across Italy) (Fig. 1). Overall, among 213 patients having a TDM-guided ECPA approach for optimizing dalbavancin treatment, 101 received long-term treatment of chronic staphylococcal infections lasting >6 weeks and were considered eligible for this study (30/101, 31.7% in the hub and 70/101, 69.3% in the spoke hospitals) (Fig. 2). Overall, 414 TDM-guided ECPAs were provided, 217 for patients receiving curative treatment (65/101, 64.4%) and 197 for those receiving suppressive treatment (36/101, 35.6%).

Demographic and clinical characteristics of patients receiving curative and suppressive treatments are summarized in Table 1. In the overall population, median (min–max) age, body mass index (BMI), and estimated glomerular filtration rate (eGFR) were 65 (12–91) years, 25.4 (16.8–48.4) kg/m^2^ and 84 (8.4–167) mL/min/1.73 m^2^, respectively. Seven patients (6.9%) had severe renal dysfunction, and two (1.98%) had eGFR ≥130 mL/min/1.73 m^2^.

**TABLE 1:** Demographic and clinical data^*a*^

Variable	Curative group (*n* = 65)	Suppressive group (*n* = 36)	*P* value
Baseline characteristics			
Age (years)	63 (48–74)	71 (53.5–79)	0.054
Gender (male/female)	45/20	22/15	0.434
Weight (kg)	75 (70–85)	70 (65–75.3)	0.079
BMI (kg/m^2^)	25.9 (24.2–28.7)	24.6 (23.1–27.7)	0.375
eGFR (mL/min/1.73 m^2^)	84.5 (64–98.7)	81.1 (59–93)	0.219
Type of infection			
Prosthetic joint infection	22 (33.8)	9 (25)	0.271
Chronic osteomyelitis	12 (18.5)	5 (13.9)	0.782
Vertebral osteomyelitis	9 (13.8)	3 (8.3)	0.320
Infective endocarditis	7 (10.8)	9 (25)	0.339
Postoperative spinal implant infection	7 (10.8)	0 (0)	0.159
Endovascular graft infection	4 (6.1)	10 (27.8)	<0.001
Septic arthritis	2 (3.1)	0 (0)	–^*e*^
Infected non-union	2 (3.1)	0 (0)	–
Microbiological isolates			
MRSE	17 (26.2)	6 (16.7)	0.499
MSSA	14 (21.5)	7 (19.4)	0.864
MRSA	7 (10.8)	8 (22.2)	0.256
MSSE	2 (3.1)	2 (5.6)	0.615
No isolate	25 (38.4)	13 (36.1)	1.000
Dalbavancin treatment			
No. of doses per patient	3 (3–4)	4 (3–10.3)	<0.001
Cumulative dose (mg)	4,500 (4,500–6,000)	6,000 (4,500–15,375)	<0.001
No. of TDM-guided ECPA per patient	3 (2–4)	4 (2–6.3)	0.005
Duration of treatment (days)	99 (77–142)	154.7 (97.8–448)^*c*^	<0.001
Clinical outcome			
Test of cure positive	51 (78.5)	NA^*d*^	0.740
Clinical success at 6-month follow-up^*b*^	17 (100)	NA	–
Adverse events	2 (3.1)	3 (8.3)	0.345

^*a*^ Continuous data are presented with median [interquartile range (IQR)] and categorical variables as count (%). BMI, body mass index; eGFR, estimated glomerular filtration rate; MRSA, methicillin-resistant *Staphylococcus aureus*; MRSE, methicillin-resistant *Staphylococcus epidermidis*; MSSA methicillin-susceptible *Staphylococcus aureus*; MSSE methicillin-susceptible *Staphylococcus epidermidis*; SSTI, skin and soft tissue infection; TDM, therapeutic drug monitoring.

^*b*^ Assessable in 17 out of 51 cases up to date.

^*c*^ Up to the last ambulatory visit available at inclusion time.

^*d*^ NA, not assessed.

^*e*^ –, absent.

Long-term dalbavancin therapy was used mainly for treating PJIs (31/101, 30.7%), endovascular prosthetic infections (14/101, 13.8%), chronic osteomyelitis (17/101, 16.8%), vertebral osteomyelitis (12/101, 11.9%), and infective endocarditis (16/101, 15.8%). Microbiological isolates were identified in 63/101 patients (62.4%). Methicillin-resistant *Staphylococcus epidermidis* (MRSE) and methicillin-susceptible *Staphylococcus aureus* (MSSA) were the most frequent ones (69.8%), followed by methicillin-resistant *Staphylococcus aureus* (MRSA; 23.8%) and methicillin-susceptible *Staphylococcus epidermidis* (MSSE; 6.3%).

The only statistically significant difference between the curative and the suppressive groups in terms of demographic, clinical, and microbiological characteristics was a higher prevalence of endovascular prosthetic infections in the suppressive group (36.1% vs 7.7%, *P* < 0.001).

**Fig 3:**
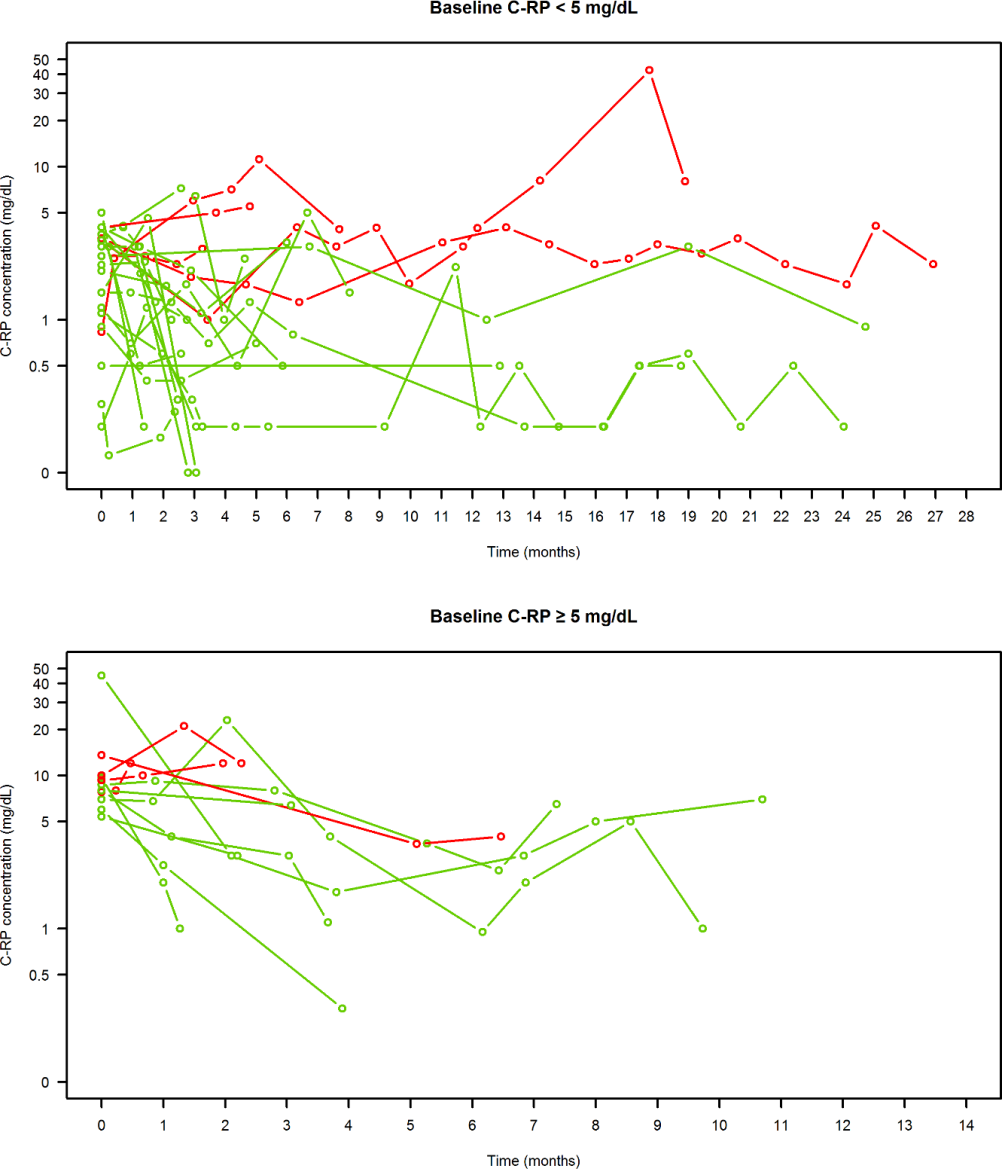
Spaghetti graph representing C-RP values overt time in each patient of the suppressive group (*n* = 36) having normal baseline C-RP value (<5 mg/dL, upper panel) or elevated baseline C-RP value (≥5 mg/dL, lower panel) in relation to clinical outcome (green = effective treatment, red = ineffective treatment).

In the curative group, the test of cure (TOC) was positive in 78.5% (51/65) of patients, and among these, the clinical success rate among cases assessable at ≥6 month follow-up up to date (17/51) was 100.0%. In the other 34/51 patients, no relapse at the median (IQR) follow-period of 2.03 (1.15–3.79) months elapsed up to date was observed. In the suppressive group, dalbavancin therapy was effective in allowing stable C-reactive protein (C-RP control in 75.0% of cases (27/36). The trend over time of C-RP values of patients in the suppressive group is shown in Fig. 3. All patients having effective suppressive dalbavancin treatment had C-RP values that remained normal or normalized after elevated baseline values.

Mild transient adverse events occurred in 5/101 patients (5.0%), two in the curative group (skin rash and shaking shiver during the third dose infusion), and three in the suppressive group (skin rash, muscle pain, and diarrhea with urticaria during the sixth dose infusion that required drug suspension).

Regression analysis of factors potentially associated with failure of dalbavancin treatment in the curative group is reported in Table 2. Interestingly, at multivariate analysis, attaining optimal PK/PD target of dalbavancin for <70% of the overall treatment duration was significantly associated with increased likelihood of treatment failure [odds ratio (OR), 6.71; confidence interval (CI), 0.97–43.33; *P* = 0.05]. Regression analysis of factors potentially associated with ineffective treatment in the suppressive group is reported in Table 3. Infective endocarditis resulted significantly associated with an increased risk of ineffective suppressive treatment with uncontrolled C-RP (OR, 8.65; CI, 1.29–57.62; *P* = 0.046), whereas overweighing only trended toward an increased risk (*P* = 0.062).

**TABLE 2:** Factors associated at regression analysis with treatment failure in patients receiving dalbavancin for long-term treatment in the curative group (*n* = 65 patients)^*a*^

Factor		Treatment outcome		Univariate analysis		Multivariate analysis	
		Cured (*n* = 51)	Failed (*n* = 14)	*P* value	OR (95% CI)	*P* value	OR (95% CI)
Age		65.0 (48.0–75.5)	61.5 (51.8–69.8)	0.555	–^*b*^	–	–
Weight		75.0 (70.0–85.0)	72.8 (70.0–83.8)	0.713	–	–	–
BMI ≥ 25		33 (64.7)	8 (57.1)	0.756	–	–	–
Gender (male)		33 (66.7)	11 (78.6)	0.520	–	–	–
eGFR		84.5 (59.5–98.4)	83.9 (69.3–98.5)	0.808	–	–	–
Duration of treatment		99.0 (78.0–148.0)	117 (73.5–139.8)	0.667	–	–	–
% of time with optimal exposure							
<70%		2 (3.9)	3 (21.4)	0.060	6.41 (0.65–85.67)	0.05	6.71 (0.97–43.33)
<80%		11(21.6)	3 (21.4)	1.000	–	–	–
<90%		22 (43.1)	5 (35.7)	0.763	–	–	–
Diagnosis							
Prosthetic joint infection		15 (29.4)	7 (50.0)	0.204	–	–	–
Chronic osteomyelitis		10 (19.6)	2 (14.2)	1.000	–	–	–
Vertebral osteomyelitis		7 (13.7)	2 (14.3)	1.000	–	–	–
Infective endocarditis		7 (13.7)	0 (0)	–	–	–	–
Endovascular graft infection		3 (5.9)	1 (7.1)	1.000	–	–	–
Patients with isolates		31 (30.8)	8 (57.1)	1.000	–		–
Type of isolate							
MRSA		5 (9.8)	3 (21.4)	0.354	–	–	–
MSSA		11 (21.6)	3 (21.4)	1.000	–	–	–
MRSE		14 (27.5)	1 (7.1)	0.159	0.21 (0.004–1.64)	0.33	2.28 (0.43–12.17)
MSSE		1 (1.9)	1 (7.1)	0.387	–	–	–
No isolate		20 (39.2)	6 (42.9)	1.000	–	–	–

^*a*^ Continuous data are presented with median (IQR) and categorical variables as count (%). BMI, body mass index; eGFR, estimated glomerular filtration rate; MRSA, methicillin-resistant *Staphylococcus aureus*; MRSE, methicillin-resistant *Staphylococcus epidermidis*; MSSA methicillin-susceptible *Staphylococcus aureus*; MSSE methicillin-susceptible *Staphylococcus epidermidis.*

^*b*^ –, absent.

**TABLE 3:** Factors associated at regression analysis with treatment failure in patients receiving dalbavancin for long-term treatment in the suppressive group (*n* = 36 patients)^*a*^

Factor		Treatment efficacy		Univariate analysis		Multivariate analysis	
		Effective (*n* = 27)	Ineffective (*n* = 9)	*P* value	OR (95% CI)	*P* value	OR (95% CI)
Age		73.0 (57.5–79.0)	63.0 (51.0–77.0)	0.486	–^*b*^	–	–
Weight		70.0 (64.0–73.0)	80.0 (70.0–84.0)	0.031	–	–	–
BMI ≥ 25		8 (29.6)	6 (66.7)	0.058	4.75 (0.95–23.8)	0.062	5.76 (0.91–36.75)
Gender (male)		15 (55.6)	6 (66.7)	0.705	–	–	–
eGFR		78.8 (58.0–92.6)	89.0 (63.0–99.0)	0.678	–	–	–
Duration of treatment		145.6 (97.2–386.9)	203.2 (100.5–562.2)	0.609	–	–	–
% of time of optimal exposure							
<70%		1 (3.7)	0 (0)	–	–	–	–
<80%		3 (11.1)	0 (0)	–	–	–	–
<90%		5 (18.5)	2 (22.2)	1.000	–	–	–
Diagnosis							
Prosthetic joint infection		6 (22.2)	3 (33.3)	0.660	–	–	–
Chronic osteomyelitis		4 (14.8)	1 (11.1)	1.000	–	–	–
Vertebral osteomyelitis		3 (11.1)	0 (0)	–	–	–	–
Infective endocarditis		4 (14.8)	5 (55.6)	0.022	7.19 (1.33–38.95)	0.046	8.65 (1.29–57.62)
Endovascular graft infection		10 (37.1)	0 (0)	–	–	–	–
Patients with isolates		16 (59.3)	7 (77.8)	0.437	–	–	–
Type of isolate							
MRSA		8 (29.6)	0 (0)	–	–	–	–
MSSA		4 (14.8)	3 (33.9)	0.333	–	–	–
MRSE		5 (18.5)	1 (11.1)	1.000	–	–	–
MSSE		1 (3.7)	1 (11.1)	0.443	–	–	–

^*a*^ Continuous data are presented with median (IQR) and categorical variables as count (%). BMI, body mass index; eGFR, estimated glomerular filtration rate; MRSA, methicillin-resistant *Staphylococcus aureus*; MRSE, methicillin-resistant *Staphylococcus epidermidis*; MSSA methicillin-susceptible *Staphylococcus aureus*; MSSE methicillin-susceptible *Staphylococcus epidermidis.*

^*b*^ –, absent.

**Fig 4:**
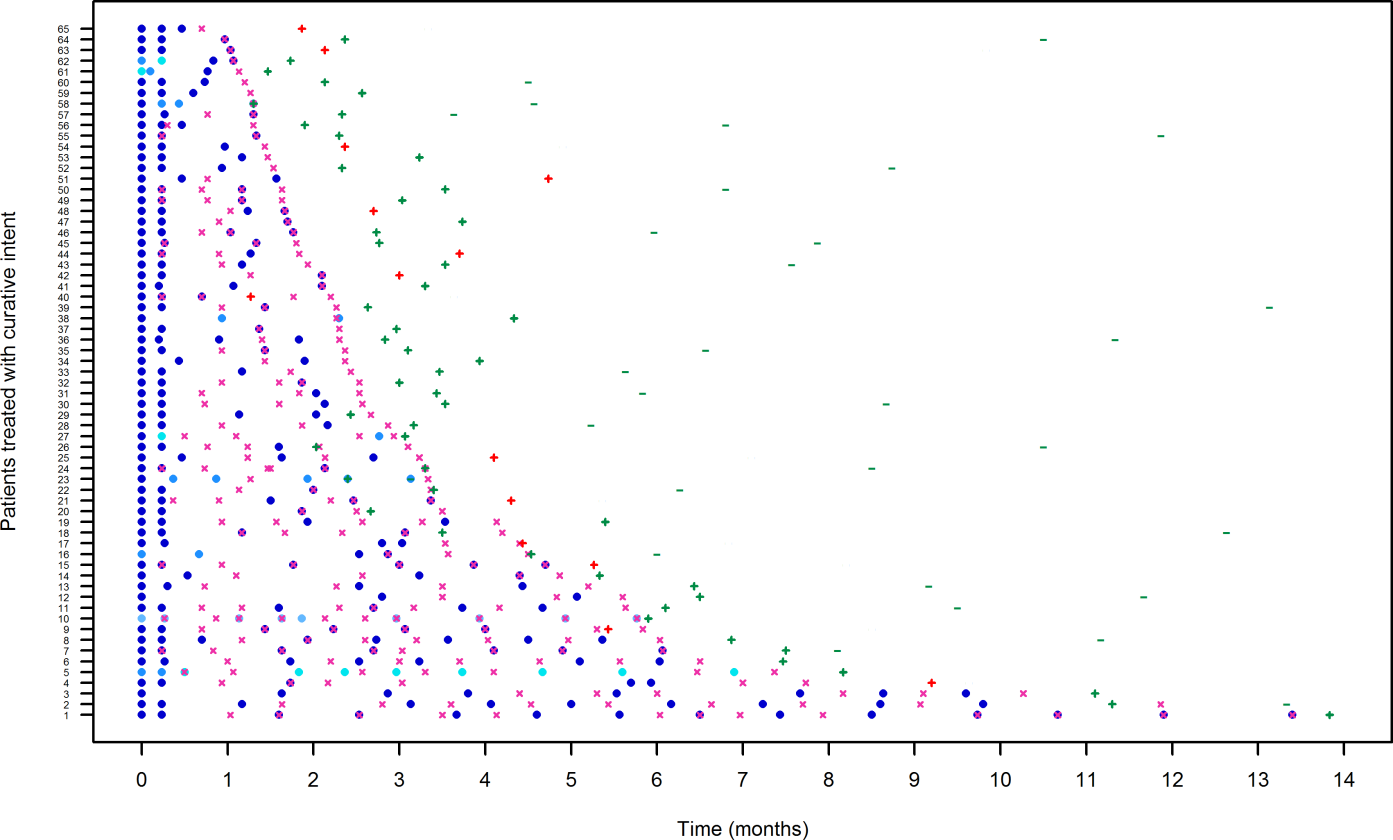
Pathway of each patient of the curative group (*n* = 65) in terms of timing and amount of administered dalbavancin doses, of provided TDM-guided expert clinical pharmacological advice (ECPA), and of clinical outcome. Dark blue, blue, sky blue, and turquoise dots represent dalbavancin dose amount of 1,500, 1,000, 800, and 500 mg, respectively; fuchsia crosses represent TDM-guided ECPA assessment; green or red + represents test of cure (TOC) positive or negative, respectively; green or red − represents a clinical successful or unsuccessful 6-month follow-up, respectively.

**Fig 5:**
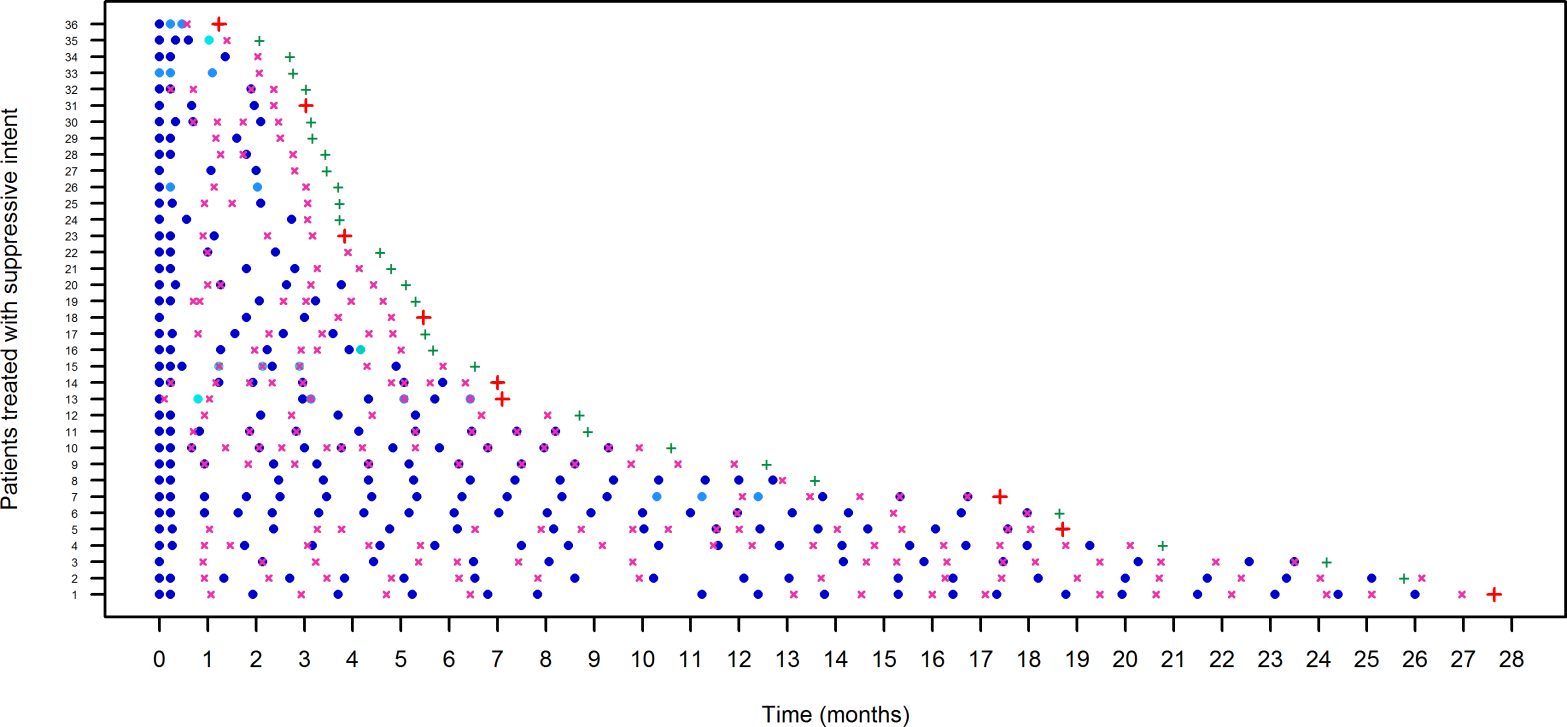
Pathway of each patient of the suppressive group (*n* = 36) in terms of timing and amount of administered dalbavancin doses, of provided TDM-guided expert clinical pharmacological advice (ECPA), and of clinical outcome. Dark blue, blue, cyan, and turquoise dots represent dalbavancin dose amount of 1,500, 1,000, 750, and 500 mg, respectively; fuchsia crosses represent TDM-guided ECPA assessment; green or red + represents dalbavancin treatment efficacy or inefficacy, respectively.

The pathway of each patient over time in terms of optimized dalbavancin treatment and of clinical outcome in the curative and in the suppressive groups are reported in Fig. 4 and 5, respectively. The inter-patient variability in terms of timing for TDM assessment and timing for re-dosing was quite high, either in the curative group [median (IQR) of 29 (25–41) days and coefficient of variation (CV) of 62.5% and median (IQR) of 27 (19–34) days and CV of 49.2%, respectively] or in the suppressive group [median (IQR) of 35 (28–47) days and CV of 107.3% and median (IQR) of 35 (25.5–49.5) days and CV of 121.1%, respectively].

**Fig 6:**
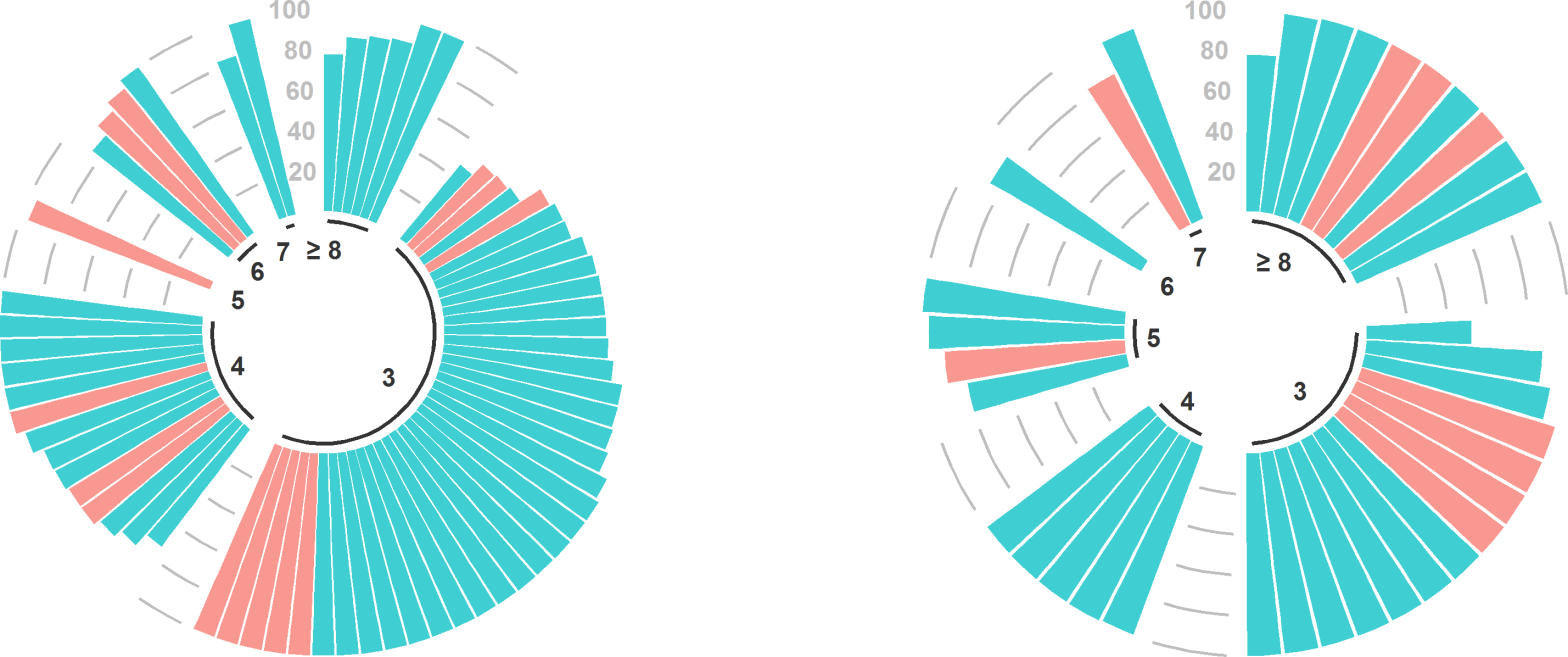
Circular barplots of the individual proportion of time with optimal PK/PD target attainment of dalbavancin during the overall treatment duration in relation to the number of dalbavancin doses and the clinical outcome in the curative group (left panel) and in the suppressive group (right panel). Green and red columns represent, respectively, test of cure (TOC) positive or negative in the curative group, effective or ineffective treatment in the suppressive group.

The proportion of time with optimal dalbavancin PK/PD target attainment in relation to the number of administered doses and to the clinical outcome is shown for each single patient in Fig. 6. The proportion of time with optimal PK/PD target attainment was high in both groups, being ≥80% in 80.0% (52/65) vs 91.7% (33/36) of cases and ≥90% in 60.0% (39/65) vs 80.5% (29/36) of cases in the curative vs the suppressive group, respectively.

## DISCUSSION

To the best of our knowledge, this is the first study assessing the role of a hub and spoke TDM-guided ECPA program of dalbavancin in the management of long-term treatment of chronic staphylococcal infections. The findings showed the usefulness that such a program may have in optimizing very long-term dalbavancin therapy. Interestingly, most patients attained very high proportions of optimal PK/PD target over time in both groups and had favorable outcome either in terms of TOC in the curative group or of effective control of the infection in the suppressive group. This may support the contention that this may be a valuable approach for providing optimal treatment with long-lasting dalbavancin therapy even to patients admitted in hospitals lacking in-house MD clinical pharmacologists.

Nowadays, optimal PK/PD target attainment is considered a fundamental requirement for antibiotic therapy either to be effective or to prevent resistance development (16, 18). This may become even more relevant with dealing with long-term treatment of chronic infections since the risk of treatment failure and of resistance development is proportional to the duration of drug underexposure. Since the beginning of dalbavancin off-label use, this aspect emerged as an unmet clinical need to be addressed for guiding properly timelines and amounts of re-dosing over time.

We first showed that keeping total dalbavancin plasma concentrations in patients above 8.04 mg/L, by ensuring optimal PK/PD target attainment against staphylococci in terms of *f*AUC_24h_/MIC ratio > 111.1 steadily over time, may be helpful for this purpose (10, 11). In this regard, we recently showed that administering two dalbavancin doses of 1,500 mg one week apart could cover up to 6 weeks of treatment in terms of optimal exposure against staphylococcal infections (12) and may be effective in leading to >90% C-RP reduction vs baseline in patients with bone and joint infections (19).

Long-term dalbavancin use may become very challenging for clinicians whenever dealing with very long treatment duration lasting several months. In this scenario, TDM-guided optimization becomes fundamental (14). A recent study showed that proactive TDM of dalbavancin was effective in facilitating a more precise targeted use of dalbavancin in the long term-management of chronic osteoarticular and periprosthetic joint infections for up to 15 month (20). It is worth noting that our patients had median duration of treatment >14 weeks in the curative group and >22 weeks in the suppressive group, with treatment lasting in some cases even more than a year. Adopting an MIPD Bayesian approach allowed our program to optimize treatment with good predictive accuracy in each single patient so that most had very high proportions of optimal exposure in both groups. This led a favorable response in the vast majority of cases, either in terms of clinical outcome in the curative group or of stable C-RP control in the suppressive group.

Noteworthy, in the curative group, having >30% treatment duration below the desired PK/PD threshold emerged as a significant risk factor of treatment failure. Suboptimal exposure observed in these cases was ascribed to poor patient's adherence in meeting the scheduled site visits and highlights how impactful an untimely management of these cases may be. Likewise, in the suppressive group, infective endocarditis was associated with the risk of ineffective control of the underlying disease. This does not surprise since source control with only antibiotic therapy without surgery is very difficult to be reached in infective endocarditis and often is unfeasible. In a small cohort of patients having long-term dalbavancin therapy as sequential treatment of cardiovascular prosthetic infection, even if the TDM-guided ECPA approach of dalbavancin increased the probability of clinical success compared to standard of care (87.5% vs 60%), inadequate source control and severity of illness were detrimental on treatment outcome (21). This is in agreement with previous findings (22) and may suggest how complex may be treating staphylococcal infective endocarditis only with suppressive antibiotic therapy (23). In this regard, the previously mentioned review reported that, in the particular context of endocarditis and cardiac-related infections, the relapse rate may be as high as 50% and that device retention was associated with higher mortality rates (9).

Finally, it might be speculated that this program could be helpful in reducing resistance development to dalbavancin during long-term use. In this regard, it was recently hypothesized that dalbavancin underexposure could have been the likely cause of dalbavancin resistance development (with a fourfold increase in MIC value from 0.032 to 0.5 mg/L) and treatment failure in a patient with *Staphylococcus epidermidis* periprosthetic joint infection receiving long-term treatment lasting 6 months (24).

We are aware of some limitation of this study. The retrospective study design and the limited sample size should be acknowledged. The heterogeneity of the patient case mix in terms of both type of infections and managing hospitals might have introduced some potential biases in patient outcome evaluation. However, criteria for outcome definition were precisely defined and applied. Conversely, the safety of this approach was very high since drug withdrawal due to adverse events occurred only in one patient during the last dose infusion, differently from what reported with other types of suppressive therapies (9).

In conclusion, our study may support the contention that a hub and spoke TDM-guided ECPA program of dalbavancin may be cost-effective for optimizing long-term treatment of chronic staphylococcal infections and for patients admitted to hospitals lacking in-house MD clinical pharmacologists or ID clinical pharmacists. A prospective confirmatory study is warranted.

## MATERIALS AND METHODS

### Study population

This multicentric retrospective cohort study was carried out in the period from April 2022 to September 2024. It included patients receiving at different Italian hospitals long-term dalbavancin monotherapy lasting >6 weeks for treating chronic staphylococcal infections and having treatment optimized by means of a TDM-guided ECPA program carried out at the Clinical Pharmacology Unit of the IRCCS Azienda Ospedaliero-Universitaria di Bologna, Italy.

Off-label indications for long-term dalbavancin monotherapy included PJI, BJI, infective endocarditis, and vascular graft infections. PJIs were identified according to EBJIS definition, in the presence of criteria for the categories “infection likely” and “infection confirmed” (25). Recommended treatment duration was of 12–24 weeks, depending on the infection site, in case of debridement antibiotics and implant retention (DAIR) intervention, and of 12 weeks in case of one-stage revision (26). Fracture-related infections (FRI) were defined as the isolation of phenotypically indistinguishable microorganisms from the culture of at least two separate deep tissue/implant specimens in the presence of a sinus tract breakdown coupled with the presence of >5 polymorphonuclear cells per high-power field at histopathology of intraoperative specimens (27). In patients with FRI, standard antibiotic treatment duration was 12 weeks (20). Infections after spinal instrumentation were defined in the presence of signs or symptoms suggestive of surgical site infection and intraoperative findings suggestive of infection and/or presence of positive intraoperative cultures. In this latter case, duration of antibiotic treatment was up to 12 weeks whenever implant was retained . In case of hematogenous vertebral osteomyelitis, usual duration of antimicrobial treatment was 6 weeks, but a longer treatment duration up to 12 weeks was considered in patients with MRSA infection perceived to be at high failure risk (28). Infective endocarditis was defined according to ESC 2015 criteria (29). Vascular graft infections diagnosis and management followed the recommendations of the American Heart Association (30).

Diagnosis of these types of chronic staphylococcal infection was based on appropriate clinical, microbiological, and imaging evaluation. Briefly, in case of bone and joint infections, patients with hematogenous vertebral osteomyelitis underwent computer-tomography-guided vertebral biopsy, and bioptic material was sent for microbiological culture; those with other types of osteoarticular infections underwent surgical debridement coupled with removal of fixation devices or prosthesis explantation whenever needed/feasible, followed by intra-operative microbiological culture. In case of endocarditis/vascular graft infections, patients underwent transthoracic and/or transesophageal echocardiography (TEE) and/or computed tomography (CT) angiography or cardiac one and/or 18F-fluorodeoxyglucose (FDG)-positron emission tomography (PET) coupled with blood culture.

Only patients receiving dalbavancin monotherapy for treating suspected or documented chronic staphylococcal infections lasting >6 weeks and optimized by means of TDM-guided ECPAs were included in this study. The study was approved by the local Ethics Committee (number 897/2021/Oss/AOUBo).

The initial standard dosing scheme was based on two 1,500 mg dalbavancin doses one week apart on day 1 and on day 8 (1,000 mg if eGFR was <30 mL/min). In the interval between day 21 and day 35 from starting treatment, all patients underwent blood sampling for TDM purposes, and after blood sample centrifugation, plasma was separated, frozen and sent to the Clinical Pharmacology Unit of the IRCCS, Azienda Ospedaliero-Universitaria di Bologna for measuring dalbavancin concentrations by means of a validated liquid chromatography-tandem mass spectrometry method (31). The method had intra- and inter-day coefficients of variation of the quality controls of 0.09–0.14% and 4.8–14.2%, respectively, and a lower limit of detection of 0.5 mg/L (31).

TDM-guided ECPAs were available twice weekly, usually on Monday and on Thursday, with a turnaround time ranging 1–5 days from sample delivery. TDM-based ECPAs were elaborated by means of a validated Bayesian method for accurately forecasting duration of optimal PK/PD target attainment, as described elsewhere (16). Briefly, TDM results, along with patient's data (age, weight, height, gender, and eGFR) and dalbavancin dosing schedule, were inserted in the software. By means of *a posteriori* approach, it was forecasted for how long total dalbavancin plasma concentration could have persisted above the threshold concentration of 8.04 mg/L. This threshold was previously identified to be useful at granting optimal PK/PD target attainment of an *f*AUC/MIC ratio > 111.1 over time against all staphylococcal strains susceptible to dalbavancin, namely, those with an MIC value up to the EUCAST clinical breakpoint of 0.125 mg/L (11). By means of this approach, the MD clinical pharmacologist located in the hub provided via internet to clinicians located either in the hub or in the spoke hospitals electronic ECPAs estimating duration of optimal exposure, timeline for subsequent TDM assessment, and/or for eventual re-dosing in case of further need for treatment prolongation.

Patients could have received dalbavancin therapy for curative purposes (curative group) or for suppressive purposes (suppressive group). In the curative group, the aim of treatment was infection healing. Patients included in this group could have had early PJIs undergoing DAIR, PJIs undergoing one-stage or two-stage revision, spinal implant infections, chronic osteomyelitis undergoing radical surgery with massive bone resection, and other osteoarticular infections or infective endocarditis. In these patients, treatment duration was defined as the time elapsed from starting dalbavancin therapy up to TOC, which was assessed repeatedly over time by means of ambulatory visits. The final visit considered TOC positive in the curative group if all of the following criteria were satisfied: absence of local (rubor, tumor, calor, dolor) and/or systemic (fever and/or pain) signs of infection coupled with normal C-RP values and absence of findings suggestive for infection at imaging studies (32). Whenever TOC was positive, dalbavancin therapy was stopped and a 6-month follow-up period was started for confirming clinical success. In the suppressive group, the aim of treatment was to improve signs and symptoms of the infection and to prevent or slow its progression. Patients included in this group could have had definitive surgical source control unfeasible, contraindication to surgery because of comorbidities and/or had refused surgery. Being these infections presumed to be incurable by means of a definitive source control, treatment duration was intended to be indefinite. In these patients, dalbavancin therapy was considered effective whenever sign and/or symptoms of infection were under control and C-RP persisted normal or normalized after baseline elevation. Failure was defined as the need of changing antimicrobial treatment and/or death.

### Statistical analysis

The Kolmogorov-Smirnov test was used to assess whether data were normally or non-normally distributed. Accordingly, means ± SD or medians with IQR were used in descriptive statistics. Statistical difference between groups was assessed by means of the chi-squared test or Fisher's exact test, when required. Univariate logistic regression analysis was used to investigate clinical factors potentially associated with treatment failure with dalbavancin in the curative and suppressive intention groups. All the independent variables associated with *P* < 0.20 at the univariate analysis were included in the multivariate model. All statistical analysis and graphs were performed with R version 4.3.3 (The R Foundation for Statistical Computing, Vienna, Austria).
